# Combining quantum cascade lasers and plasmonic metasurfaces to monitor *de novo* lipogenesis with vibrational contrast microscopy

**DOI:** 10.1515/nanoph-2025-0014

**Published:** 2025-08-27

**Authors:** Steven H. Huang, Dias Tulegenov, Gennady Shvets

**Affiliations:** School of Applied and Engineering Physics, 5922 Cornell University, Ithaca, NY 14850, USA

**Keywords:** metasurface, surface-enhanced infrared absorption, infrared microscopy, *de novo* lipogenesis, adipocytes

## Abstract

The combination of a tunable quantum cascade laser (QCL) and plasmonic mid-infrared (MIR) metasurface is a powerful tool enabling high-content microscopy of hydrated cells using the vibrational contrast of their constituent biomolecules. While the QCL provides a high-brightness source whose frequency can be rapidly tuned to that of the relevant molecular vibration, the metasurface is used to overcome water absorption of MIR light. Here we employ the resulting metasurface-enabled inverted reflected-light infrared absorption microscopy (MIRIAM) tool for non-destructive monitoring of the vital process of *de novo* lipogenesis (DNL), by which fat tissue cells (adipocytes) synthesize fatty acids from glucose and store them inside lipid droplets. Using ^13^C-labeled glucose as a metabolic probe, we produce spatially- and temporally-resolved images of ^13^C incorporation into lipids and proteins, observed as red-shifted vibrational peaks in the MIR spectra. These findings demonstrate MIRIAM’s capability for studying metabolic pathways with molecular specificity, offering a powerful platform for metabolic imaging.

## 1 Introduction

Among the many contributions of Federico Capasso to science, two especially stand out for the biophotonics community: the invention of the quantum cascade laser (QCL) [1] and the pioneering work in the area of mid-infrared (MIR) plasmonic metasurfaces [2]. The MIR spectral range is particularly important for life science applications because it overlaps with the vibrational frequencies of many chemical groups prevalent in biomolecules. For example, vibrational features within the 1,500 cm^−1^ < *ν* < 1,750 cm^−1^ MIR spectral window correspond to a combination of C=O stretching, N–H bending, and C–N stretching in the amide backbone of proteins, as well as C=O stretching of the ester group in lipids [3]. Consequently, the ability to rapidly tune the emission wavelength (*λ* ≡ 1/*ν*) of a QCL to match various vibrational fingerprints of cellular molecular constituents (e.g., proteins, lipids, carbohydrates, and nucleic acids) has become highly attractive for label-free spectroscopy of cells and tissues, where the vibrational modes serve as endogenous image contrast [4], [5], [6], [7], [8], [9].

Today, major manufacturers of MIR instrumentation (e.g., Agilent, Bruker, Daylight Solutions) have integrated QCLs into their MIR microscopes, offering QCL-driven discrete-frequency infrared (DFIR) microscopes on the market, and MIR micro-spectroscopy – using either broadband incoherent light sources for Fourier transform infrared (FTIR) spectroscopy or coherent ones, such as QCLs – has become a popular tool for analyzing biological samples [10], [11], [12], [13]. Commercial DFIR microscopes have been used to analyze thin tissues section, dried biofluids, and fixed/dried cells [7], [8], [14], [15]. However, extending MIR spectroscopy to live (hydrated) cells and tissues has been hindered by the strong attenuation of MIR light in water. The use of thin flow cells and attenuated total reflection (ATR) geometries [16], [17], [18], [19], [20] have mitigated this issue to some extent but come with limitations, such as incompatibility with microwell-based cell analysis and automated liquid handling necessary for high-throughput sample analysis [21], [22].

**Figure 1:**
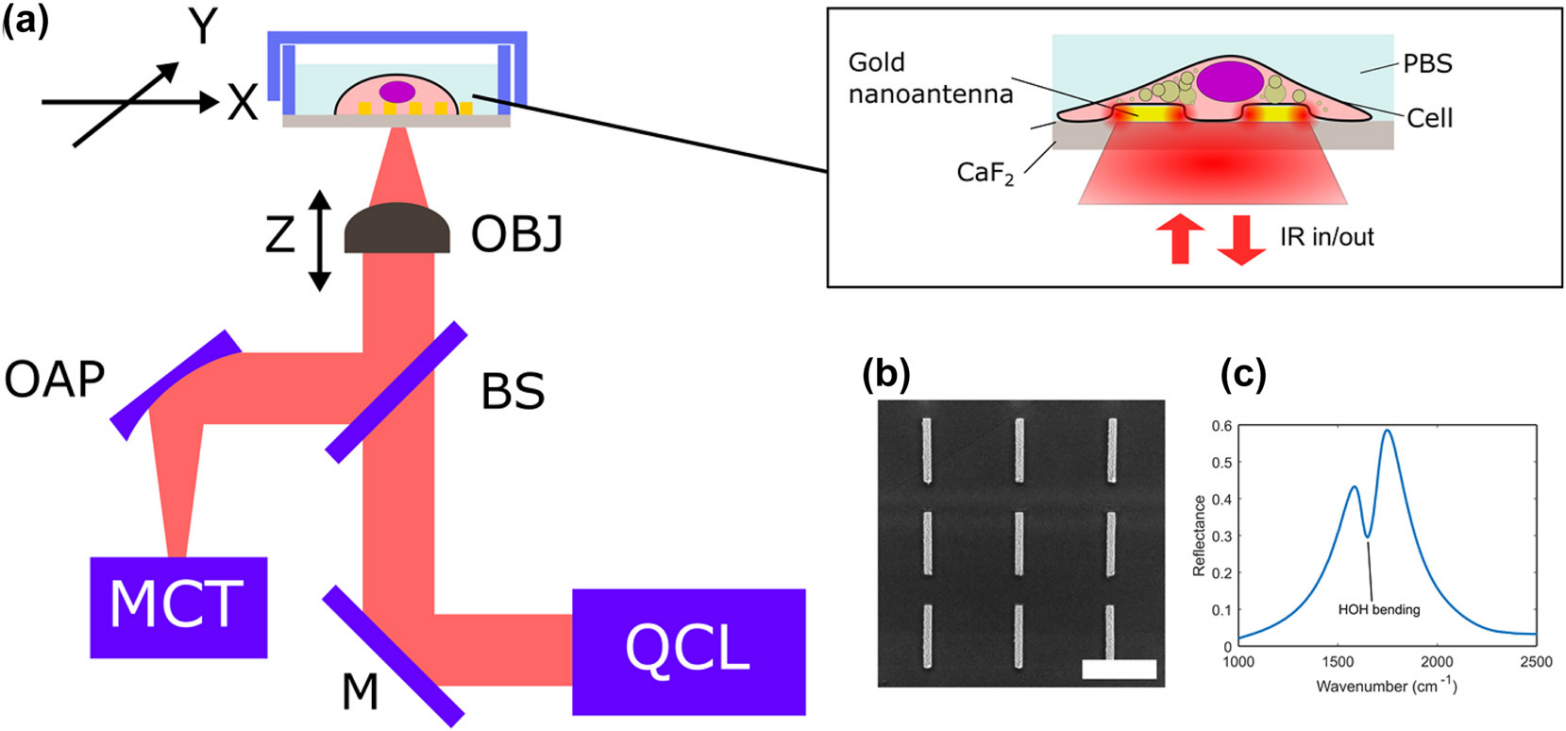
Metasurface-enabled inverted reflected-light infrared absorption microscopy (MIRIAM). (a) Schematic drawing of the experimental setup. OBJ: objective, OAP: off-axis parabolic mirror, BS: beam splitter, M: mirror, MCT: mercury–cadmium–telluride IR detector, QCL: quantum cascade laser. (b) Scanning electron microscope image of the plasmonic nanoantennas. Scale bar: 2 µm. (c) Reflectance spectrum of the metasurface in water, measured by FTIR. The dip at 1,650 cm^−1^ corresponds to H–O–H bending mode of water.

To address these limitations, we have recently demonstrated that MIR plasmonic metasurfaces [23], [24], [25], [26], [27], [28] supporting surface-enhanced infrared absorption (SEIRA) [29], [30] can be an effective tool to probe the vibrational spectra of living cell samples. Among the different geometries of nanoantennas for SEIRA (e.g., cross-shaped [31], elliptical [32], Fano-resonant asymmetric [28]), linear dipole nanoantennas are particularly popular and can be easily tuned to vibrate at a specific frequency of interest by varying its length [33], [34]. The plasmonic resonance produced by such antennas can be further enhanced through the collective resonances of the periodical nanoantenna array, achieving even stronger near-field localization and narrower resonance linewidth [33]. When applied to SEIRA spectroscopy of cellular samples, metasurfaces serve two essential functions: (i) they provide strongly localized near-field enhancements (within 100 nm from the surface) of optical fields, and (ii) they produce strong reflection signals that can be collected using an inverted infrared microscope operating in reflection mode [35] (see Figure 1). Cells are cultured on a metasurface comprising an array of plasmonic nanoantennas fabricated on an IR-transparent CaF_2_ substrate. The metasurface reflectance spectrum is modulated by interactions between the cells and the plasmonic near-field via SEIRA and these interactions are probed in the far field by measuring the reflectance spectrum. This interaction can be explained through Fano resonances where the weak absorber (dark mode) couples to the plasmonic resonance (bright mode) of the nanoantennas, and the modulation is manifested as either dip or peak in reflectance [36]. When combined with an FTIR spectrometer, these metasurfaces reveal rich molecular information and temporal dynamics about biomolecules in the plasma membrane and cytoskeleton.

While our earlier work focused on FTIR-based MIR spectroscopy of live cells, effectively averaging the MIR spectra over many cells on top of the metasurface (typically a square area with several hundred micrometers in width), being able to image at single-cell resolution is highly desirable due to the inherent heterogeneity of live cells in physiologically relevant samples. To that end, we recently developed metasurface-enabled inverted reflected-light infrared absorption microscopy (MIRIAM), a hyperspectral platform for live-cell imaging with diffraction-limited resolution using QCL as the light source [37]. Previously, we demonstrated MIRIAM’s capabilities for vibrational microscopy of cells, detecting molecular contrasts from phosphates, proteins, and lipids, which allowed clear visualization of sub-cellular structures like nuclei and lipid droplets (LDs). Unlike conventional IR microscopy, MIRIAM overcomes the attenuation of IR light in water by confining the sensing volume to a few hundred nanometers around plasmonic hotspots. The surface sensitivity of MIRIAM, on the order of ∼100 nm, is analogous to that of total internal reflection fluorescence (TIRF) microscopy [38]. Both techniques are well-suited for probing cellular features near the plasma membrane, such as cytoskeletal organization and cell-substrate adhesion. However, MIRIAM offers a distinct advantage by relying on molecular vibrational contrast, which enables cellular imaging without the need for fluorescent labels. The planar metasurface design and reflection-mode imaging facilitate straightforward integration with standard cell culture chambers, making MIRIAM suitable for high-throughput formats.

A promising application of MIR microscopy, including MIRIAM, is in the non-pertubative, all optical imaging of metabolic processes in cells (e.g., protein turnover and synthesis, or *de novo* lipogenesis (DNL)) [39], [40], [41], [42], [43], [44], [45], as metabolomics provides a readout closest to the phenotype [46], [47]. Studying cellular metabolism presents unique challenges since small molecules like glucose and lipids cannot be labeled with fluorescent tags without disrupting their metabolic roles [46]. Stable isotope labeling with ^2^H and ^13^C, combined with analytical techniques like mass spectrometry or nuclear magnetic resonance, has become a widely used approach for metabolic studies [39], [48], [49], [50], [51]. However, these methods are inherently destructive and often lack spatial resolution – although imaging mass spectrometry can provide spatial information, it requires expensive instrumentation and typically involves long acquisition times. Vibrational spectroscopy and imaging techniques, such as Raman microscopy, infrared (IR) microscopy, and mid-IR photoacoustic microscopy, have gained attention as non-destructive, spatially resolved tools for analyzing biomolecules labeled with stable isotopes [8], [45], [52]. These approaches enable fluorescence-free analysis with molecular specificity, and various labeling strategies – including ^13^C and ^2^H isotopes and probes with small azide or alkyne functional groups – have been developed for metabolic studies [8], [53]. Despite their promise, these techniques have limitations for cellular studies. For instance, Raman spectroscopy suffers from low signal intensity due to the small Raman cross-section of biomolecules, often necessitating high laser power, which can lead to photodamage and phototoxicity [54]. Similarly, mid-IR photothermal microscopy, while offering sub-500 nm spatial resolution comparable to optical microscopy, has a compromised signal-to-noise ratio when used with aqueous samples due to water’s small thermo-optic coefficient [52], [55], [56].

In this manuscript, we explore the application of the MIRIAM platform for characterizing metabolic processes, focusing on monitoring DNL in adipocytes (fat tissue cells). DNL – the process by which cells synthesize fatty acids from non-lipid precursors like glucose – is a crucial pathway in adipocyte biology and systemic metabolic health [57], [58]. In adipocytes, DNL provides the basis for energy storage in the form of triglycerides, linking glucose metabolism to lipid synthesis via the tricarboxylic acid (TCA) cycle and subsequent lipogenic pathways. Dysregulation of DNL has been implicated in metabolic disorders such as obesity and type 2 diabetes, making adipocyte metabolism a promising target for cardiometabolic disease treatments.

In this work, we used MIRIAM to investigate DNL in mouse adipocyte cells using ^13^C-glucose as an IR-active probe. When substituted for ^12^C-glucose in the culture medium, ^13^C-glucose is metabolized into lipids stored in LDs. While the penetration depth of MIRIAM is limited, many – but not all – lipid droplets remain detectable. The heavier ^13^C isotope induces a redshift in vibrational modes compared to ^12^C, detectable in IR spectra. We demonstrate, through MIRIAM imaging of fixed adipocytes in buffer environment, that ^13^C incorporation into lipids and proteins is readily visualized with MIRIAM, reflecting glucose metabolism rates. Our findings highlight MIRIAM’s imaging capabilities and its sensitivity to ^13^C as a metabolic label, paving the way for parallel use of multiple metabolic labels to uncover intricate metabolic profiles through image-based cellular analyses.

## 2 Experimental

### 2.1 Metasurface fabrication

The plasmonic nanoantennas were fabricated using electron beam lithography (EBL) on calcium fluoride (CaF_2_) substrate using PMMA resist. A 5 nm chromium adhesion layer and a 70 nm gold layer were deposited sequentially using thermal evaporation. The plasmonic nanoantennas were then formed by a lift-off process in acetone. The resulting metallic nanoantennas were arranged in an array covering a 500 µm × 500 µm area, constituting the metasurface. To enable imaging in aqueous environments, the metasurface was attached to the bottom of a cell culture chamber superstructure (CS16-CultureWell Removable Chambered Coverglass, Grace Bio-Labs).

### 2.2 Cell culture, differentiation, and ^13^C labeling

Undifferentiated mouse embryonic fibroblast 3T3-L1 cells (American Type Culture Collection) were cultured in pre-adipocyte expansion medium consisting of Dulbecco’s Modified Eagle Medium (DMEM, Gibco) with 4.5 g/L glucose and GlutaMAX supplement, 10 % fetal bovine serum (FBS, Gibco), and 1 % penicillin/streptomycin (P/S, Gibco). Cells were maintained at 37 °C in a 5 % CO_2_ incubator.

Upon reaching confluency, cells were incubated for another 48 h before switching to differentiation medium. The differentiation medium consisted of DMEM supplemented with 10 % FBS, 1 % P/S, 1 μM dexamethasone (Sigma-Aldrich), 500 μM 3-isobutyl-1-methylxanthine (IBMX, Sigma-Aldrich), 2 μM rosiglitazone (Sigma-Aldrich), and 2 μg/mL bovine insulin (Sigma-Aldrich) [59]. After 48 h in differentiation medium, the cells were transitioned to adipocyte maintenance medium, composed of DMEM supplemented with 10 % FBS, 1 % P/S, and 2 μg/mL bovine insulin, and cultured for an additional 5 days.

Then, cells were harvested using 0.25 % trypsin-EDTA (Gibco) and re-seeded onto metasurfaces. Prior to seeding the cells, the metasurface was sterilized using 70 % ethanol and coated with 0.2 % gelatin, treated overnight in the incubator in PBS. Cells were divided into eight samples, each exposed to varying durations of ^13^C glucose maintenance medium and ^12^C glucose maintenance medium. Samples were labeled day 0 to day 7, corresponding to the number of days in ^13^C glucose maintenance medium. For the ^12^C glucose maintenance medium, regular DMEM was used, while for the ^13^C glucose maintenance medium, glucose-free DMEM was supplemented with 4.5 g/L U-^13^C_6_ glucose (Cambridge Isotope Laboratories) and the same concentrations of FBS, P/S, and insulin as in the ^12^C medium. Throughput the experiment, media were exchanged to fresh media containing either ^12^C glucose or ^13^C glucose at least every 2 days.

Seven days after seeding on the metasurfaces, cells were fixed with 4 % formaldehyde for 15 min, washed three times with phosphate buffered saline (PBS), and stored in PBS prior to IR measurements.

### 2.3 Inverted MIR microscope setup

The inverted MIR microscope used in this study has been described in detail elsewhere [37]. Briefly, the MIR beam from a quantum cascade laser (QCL, MIRcat-1400, Daylight Solutions) was focused onto the metasurface from below using a Black Diamond-2 infrared collimation lens with a numerical aperture (NA) of 0.71 (390093, LightPath Technologies). The focal spot size was approximately 5 µm. The reflected beam from the metasurface was collected by the same objective lens and detected by a liquid-nitrogen-cooled MCT detector (MCT-13-1.0, InfraRed Associates).

The output current from the MCT detector was sent to a lock-in amplifier (SR830, Stanford Research Systems) for signal demodulation. For imaging, the metasurface sample was mounted on a dual-axis motorized microscope stage (HLD117, Prior Scientific), and point-scanning was performed by moving the sample through the laser focus. Hyperspectral image cubes were acquired by collecting discrete frequency images in the range of 1,500–1,800 cm^−1^ at 5 cm^−1^ intervals, with 2 µm pixel size.

To reduce water vapor absorption in the MIR, the entire optical setup was enclosed in an optical enclosure purged with dried air.

### 2.4 Data processing and analysis

The acquired images were first processed using translational image registration in ImageJ to correct for sample drift and laser pointing fluctuations. Further data processing was performed in MATLAB. To correct for detector non-linearity, the signal intensity at each pixel was adjusted using a pre-measured calibration curve. Fluctuations in laser intensity were accounted for by mean-centering each row of the image.

Absorbance was calculated using the formula:

(1) $$A\left(x,y,\omega \right)=-\log_{10}\left(R\left(x,y,\omega \right)/R\left(x_{0},y_{0},\omega \right)\right)$$

where $R\left(x,y,\omega \right)$ is the reflectance at a given pixel $\left(x,y\right)$ and wavenumber *ω*, and $R\left(x_{0},y_{0},\omega \right)$ is the reflectance of a bare metasurface at a cell-free location. The hyperspectral image was subsequently de-noised using a minimum noise fraction (MNF) transform, retaining the first 20 components of the data for further analysis.

To quantify the intensity of specific vibrational peaks, a linear baseline subtraction method was applied to correct for shifts in the absorbance baseline caused by metasurface resonance variations. Two baseline points were selected on either side of each peak for the baseline correction. For the amide II peak, the absorbance at 1,555 cm^−1^ was used to calculate the intensity, with baseline points at 1,500 cm^−1^ and 1,600 cm^−1^. For the ^13^C=O ester peak, the absorbance at 1,705 cm^−1^ was used, with baseline points at 1,685 cm^−1^ and 1,720 cm^−1^. For the ^12^C=O ester peak, the absorbance at 1,745 cm^−1^ was used, with baseline points at 1,725 cm^−1^ and 1,760 cm^−1^.

Additionally, the ^13^C=O ester peak exhibited significant overlap with the amide I peak of proteins and the H–O–H bending mode of water [8], [52]. To correct for these overlaps, the following model was used:

(2) $$B_{13\text{C }=\text{ O}}=A_{13\text{C }=\text{ O}}-aA_{\text{amide}\text{II}}-b\left(A_{12\text{C }=\text{ O}}+A_{13\text{C }=\text{ O}}\right)$$

where *B*_13C=O_ is the apparent peak intensity calculated using the linear baseline method, and *A*_13C=O_, *A*_amideII_, and *A*_12C=O_ are the true peak intensities without spectral overlap. This model is based on two assumptions: (i) the spectral overlap in the amide II and ^12^C=O ester peaks is negligible; (ii) the overlap from the amide I peak is proportional to the number of amide groups whose combined bond strength is proportional to *A*_amideII_, and the overlap from the H–O–H bending mode arises from the total lipid-associated displacement of water proportional to $\left(A_{12\text{C }=\text{ O}}+A_{13\text{C }=\text{ O}}\right)$ .

The coefficients *a* = 0.21 and *b* = 0.045 were empirically determined. The coefficient *a* was chosen to ensure that *A*_13C=O_ was zero in cytosolic regions lacking LDs, while *b* was selected such that *A*_13C=O_ was zero in LDs from the day 0 sample, which was cultured entirely in ^12^C glucose medium.

## 3 Results

### 3.1 MIRIAM imaging of adipocyte cells

MIR hyperspectral images of 3T3-L1 adipocyte cells were acquired using MIRIAM, a platform previously developed by our group [37]. A schematic of the experimental setup is shown in Figure 1a. The metasurface used in this study comprised an array of gold nanoantennas with dimensions of 200 nm in width, 1.8 µm in length, and 2.7 µm periodicity in both horizontal and vertical directions (Figure 1b). When excited with incident light polarized along the long axis of the nanoantennas, the metasurface exhibited a resonance mode at approximately 1,650 cm^−1^, with a pronounced peak in reflectance (Figure 1c). The resonance, characterized by a full-width at half maximum (FWHM) of 300 cm^−1^, covered multiple vibrational modes within the 1,500–1,800 cm^−1^ range. This single-resonance metasurface design was employed to probe key vibrational modes associated with adipocytes, including amide II (1,555 cm^−1^) and amide I (1,655 cm^−1^) for proteins, and ^13^C=O ester (1,705 cm^−1^) and ^12^C=O ester (1,745 cm^−1^) for lipids.

**Figure 2:**
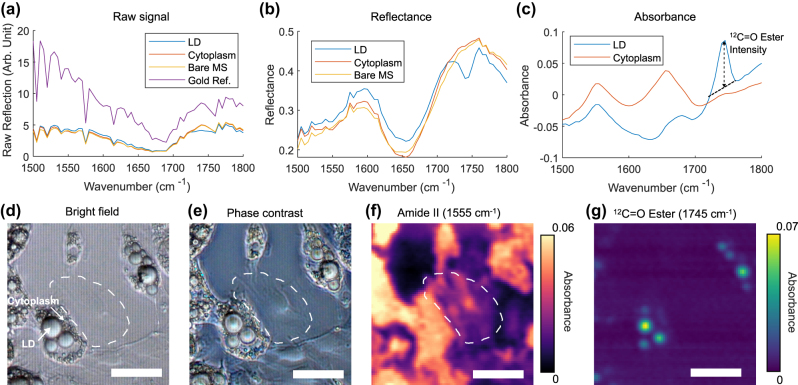
MIR spectra and vibrational contrast images of 3T3-L1 adipocytes acquired through MIRIAM. (a) Raw reflection signal at different locations. “Bare MS” refers to a metasurface region without cells, while “Gold Ref.” indicates a gold patch used for reflectance reference measurements. Cytoplasm and LD spectra are obtained at positions indicated by the arrow in (d). Sharp peaks in the spectra correspond to atmospheric water vapor absorption. (b) Reflectance spectra at different positions. (c) Absorbance spectra at LD and cytoplasm position. The black dotted line shows the linear baseline correction used to determine the ^12^C=O ester intensity. (d) Bright field image of 3T3-L1 cells on the metasurface. White dashed curve indicates the boundary of an undifferentiated fibroblast cells, barely visible in bright field image. (e) Phase contrast image of 3T3-L1 cells on the metasurface. (f) MIRIAM image at amide II band (1,555 cm^−1^). (g) MIRIAM image at ^12^C=O ester band (1,745 cm^−1^). Scale bar: 100 µm.

Figure 2a–c displays the raw signal, reflectance spectra, and absorbance spectra, respectively, from LD and cytoplasm within a cell. The positions at which the spectra are obtained are shown in Figure 2d, with a representative bright field image of fixed 3T3-L1 cells on the metasurface. LDs within the cells are clearly visible due to the high refractive index contrast between lipids and water. The gold nanoantennas of the metasurface appear as a periodic pattern in the background.

The intensity variation in the raw signal spectra (Figure 2a) results from the wavelength-dependence of QCL emission (slow variation) and atmospheric water vapor absorption (sharp dips). These variations are normalized by collecting a reference spectrum from a 70 nm thick gold patch adjacent to the metasurface, yielding the reflectance spectra shown in Figure 2b. Although our QCL-based measurement lacks the spectral range to fully capture the metasurface’s resonance mode (see FTIR measurement in Figure 1c for the full reflectance spectrum), variations in reflectance due to the metasurface resonance and a dip corresponding to the H–O–H bending mode of water are evident.

To isolate the vibrational signature of the cells, absorbance spectra are calculated using a reference spectrum collected from a bare metasurface position (Equation (1)). Note that since bare metasurface (in PBS) is used as a reference, all absorbance spectra shown in this work are differential absorbance from this reference. The absorbance spectra reveal vibrational modes corresponding to cellular biomolecules, including the amide I (1,655 cm^−1^) and amide II (1,555 cm^−1^) modes from cytoplasmic proteins, as well as the ^12^C=O ester mode (1,745 cm^−1^) from lipids in the LDs (Figure 2c). In MIRIAM, the vibrational signals of analytes are superimposed on the metasurface resonance spectrum. This results in a varying baseline in the absorbance spectrum due to shifts in the metasurface resonance when analytes interact with the near-field. To quantify the vibrational signals, a linear baseline subtraction was applied, as described in the Experimental section. Figure 2c shows the baseline subtraction for ^12^C=O ester mode, as an example.

MIRIAM vibrational contrast images of 3T3-L1 adipocyte cells in the amide II and ^12^C=O ester bands are shown in Figure 2f and g, with the corresponding bright field and phase contrast images shown in Figure 2d and e. The amide II channel, derived from the protein-associated vibrational contrast, exhibits distinctly different spatial distribution compared to the ^12^C=O ester channel derived from the lipids-associated contrast. This demonstrates the specificity of MIRIAM for detecting distinct molecular vibrations with minimal crosstalk between channels. Owing to the limited penetration depth (∼100 nm) of the plasmonic near-field surrounding the antennas, the protein signal predominantly originates from the focal adhesion sites and the cytoskeletal layer around the cell cortex. Protein contrast highlights cells spreading on the metasurface, with individual cells clearly discernible. Often, there are undifferentiated fibroblast cells mixed with the adipocyte cells. The fibroblast cells can be thinly spread (Figure 2d–f, white dashed curve), making them difficult to identify with brightfield (Figure 2d) or phase contrast microscopy (Figure 2e). These cells are clearly visible with MIRIAM in the amide II channel (Figure 2f), highlighting the excellent surface sensitivity of MIRIAM. The noise in the MIRIAM images is estimated to be 0.001 in absorbance unit, based on the standard deviation in a cell-free region. This results in a typical contrast-to-noise ratio in the range of 20–40 in the amide II channel, depending on the adhesion strength of each cell.

The signal from ^12^C=O ester channel primarily originates from lipids in the LDs. However, many LDs visible in bright field microscopy (Figure 2d) are not seen in the MIRIAM images. This is attributed to the surface sensitivity of MIRIAM, which renders LDs positioned far from the metasurface essentially invisible.

Although previous studies have demonstrated MIRIAM’s capability to image living cells at a few discrete frequencies [37], the current acquisition speed of ∼5 min for a 500 µm × 500 µm image makes hyperspectral imaging of living cells impractical, especially when collecting data for 10 or more frequencies. Movement of living cells during the measurement introduces spectral inaccuracies, particularly for small structures like LDs. To address this limitation, this study focused on imaging fixed cells while keeping the cells hydrated in PBS to avoid disruptions in cellular structures and morphology.

**Figure 3:**
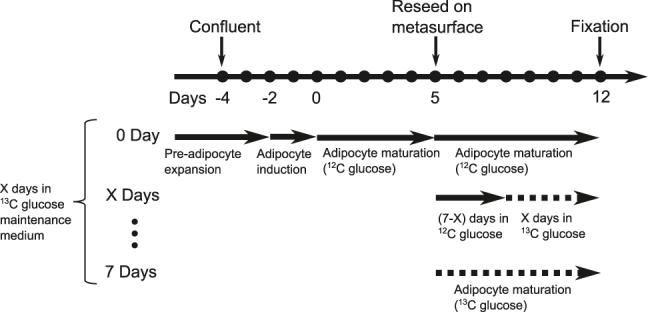
Experimental timeline for differentiation and ^13^C glucose feeding of 3T3-L1 adipocytes.

To study the glucose metabolism and DNL in 3T3-L1 adipocytes, eight samples of 3T3-L1 adipocyte cells were prepared with varying exposure times to ^13^C glucose (Figure 3). Preadipocyte cells were chemically induced to differentiate into adipocytes and cultured for 5 days in a flask until LDs were visibly formed. At this point, the cells were reseeded onto metasurfaces and incubated in maintenance media with differing exposure times to ^13^C glucose (0–7 days). This design ensured that each sample are cultured in the adipocyte maintenance medium for the same amount of time and form LDs with comparable sizes, while varying the relative amounts of ^13^C=O ester and ^12^C=O ester in LDs due to differential exposure to ^13^C glucose and ^12^C glucose.

**Figure 4:**
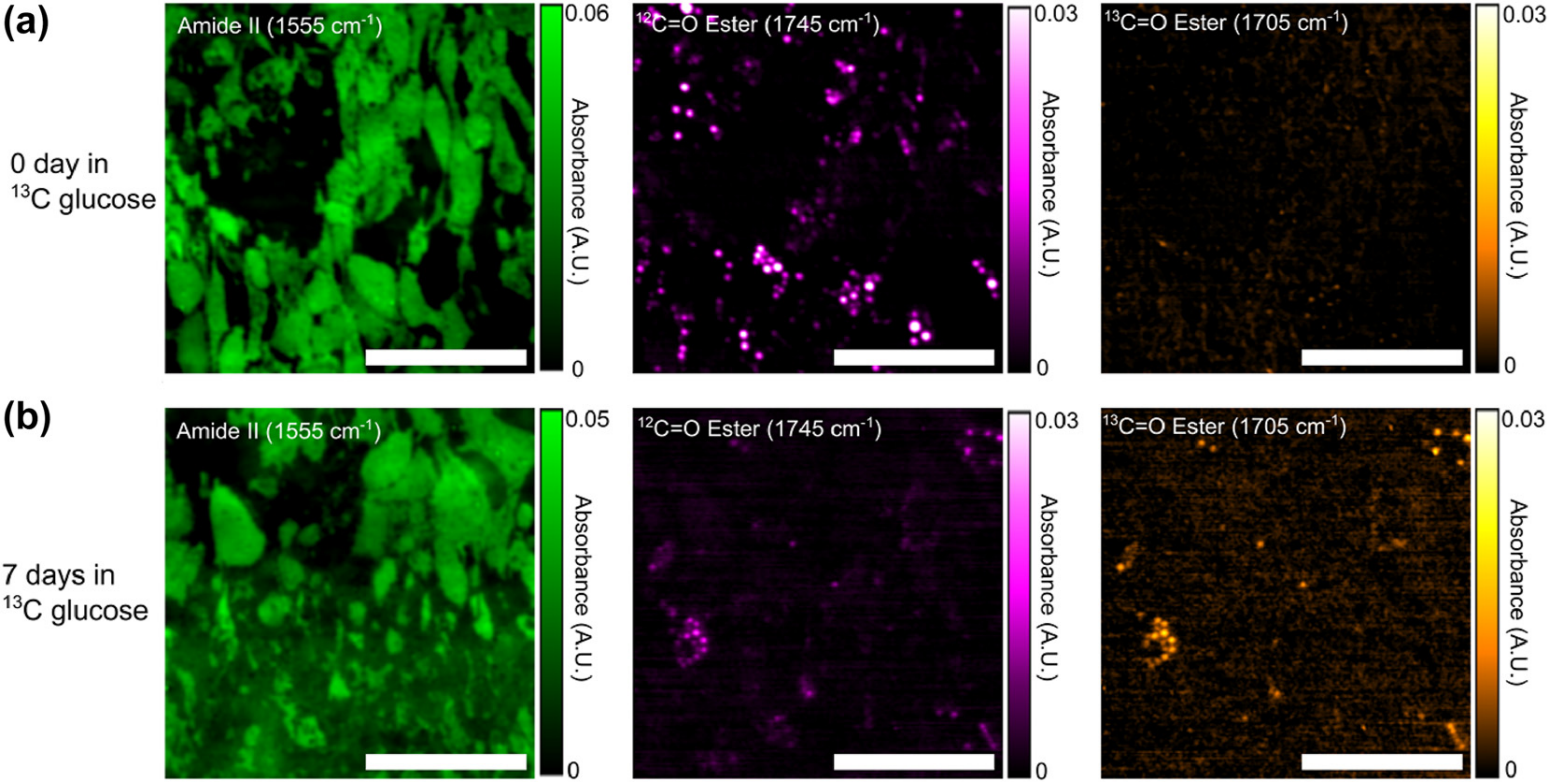
Fixed 3T3-L1 adipocyte IR absorbance images acquired by MIRIAM. (a) 0-day exposure to ^13^C glucose and (b) 7 days exposure to ^13^C glucose. Absorbance images are presented with contrast in amide II (left column), ^12^C=O ester (middle column), and ^13^C=O ester (right column) of (a, b). Increase in ^13^C=O ester and decrease ^12^C=O ester in the 7 days sample can be clearly seen in (b). Scale bar: 200 µm.

Representative MIRIAM-based MIR images of samples with 0 days (no exposure) and 7 days of ^13^C glucose exposure are shown in Figure 4, and the corresponding bright field images are presented in Figure S2. Despite the limited visibility of LDs due to the shallow penetration depth of metasurface, sufficient LDs were visible to quantify ^12^C=O and ^13^C=O ester signals. In the 0-day sample, LDs exhibited no detectable ^13^C=O ester signal, as expected, with all lipids derived from ^12^C glucose. In contrast, the 7-day sample showed a marked increase in the ^13^C=O ester signal and a corresponding decrease in the ^12^C=O ester signal within LDs. The colocalization of ^13^C=O and ^12^C=O signals confirmed that both signals originated from the same LDs, with the ratio reflecting the time-dependent increase of ^13^C incorporation into the LDs with prolonged ^13^C glucose exposure.

We note that the combination of the inverted QCL-based microscope and the metasurface enabled diffraction-limited imaging of sub-cellular LDs inside hydrated cells. Earlier attempts at spectrochemical imaging using commercial widefield MIR microscopes worked exclusively with dried samples and encountered coherence-related speckle artifacts in transmission-mode images [7], [8]. By using a plasmonic metasurface in addition to a widefield MIR microscope, Rosas et al. demonstrated enhanced MIR spectra of dried tissue samples in both transmission and reflection mode, but no applications to hydrated samples were reported [9]. Likewise, confocal MIR microscopy without a metasurface produced speckle-free vibrational images with diffraction-limited resolution, but only in the transmission mode [5], [6], which cannot be used for imaging hydrated cells.

### 3.2 Tracking ^13^C incorporation in lipids and proteins through IR spectra

**Figure 5:**
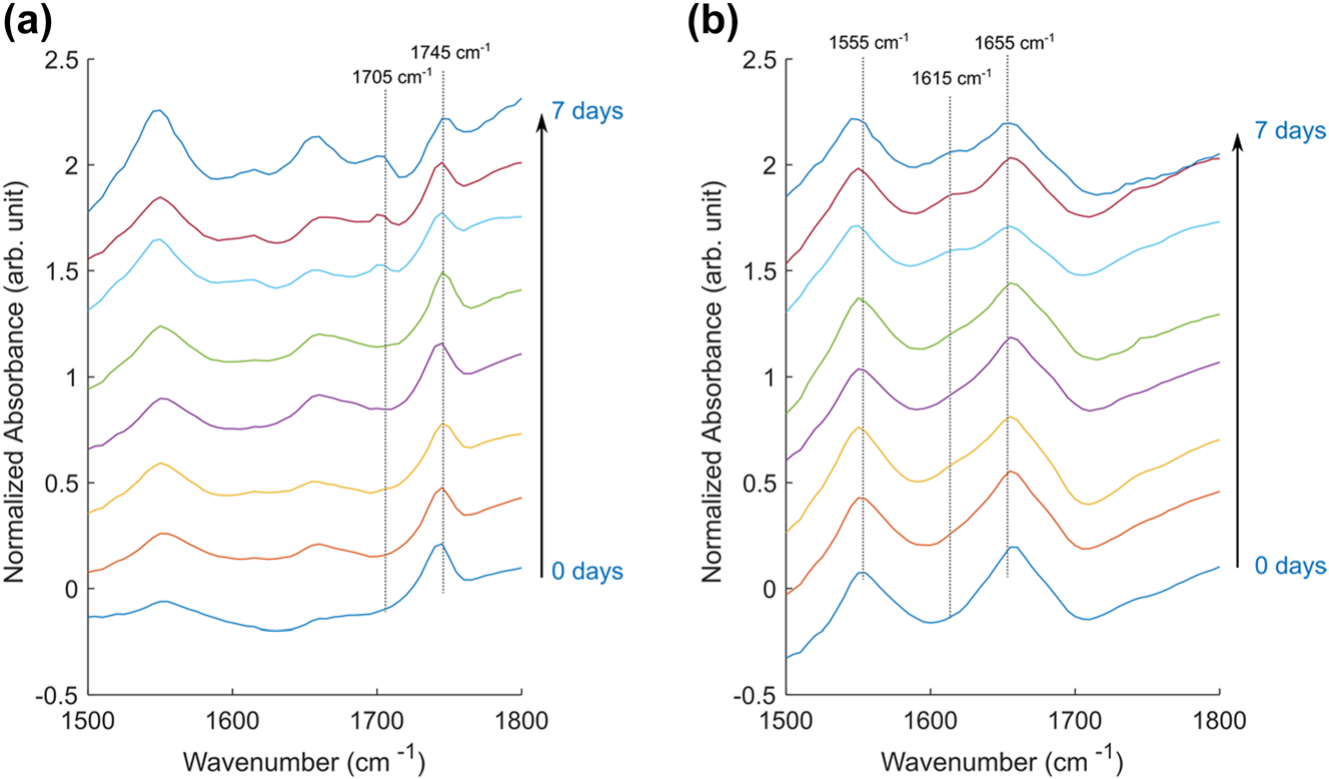
Mean IR spectra from (a) LDs and (b) cytoplasm regions without LDs, for samples with varying exposure times to ^13^C glucose. Increases in the ^13^C=O ester peak (1,705 cm^−1^) and the ^13^C amide I peak (1,615 cm^−1^) are observed with longer exposure to ^13^C glucose medium.

To investigate the temporal evolution of ^13^C glucose metabolism, mean absorbance spectra were extracted from each sample (Figure 5), focusing on two distinct regions: LDs (Figure 5a) and the cytoplasmic region outside of the LDs (Figure 5b).

In the LD spectra, increasing exposure time to ^13^C glucose led to a pronounced increase in the ^13^C=O ester peak at 1,705 cm^−1^ and a corresponding decrease in the ^12^C=O ester peak at 1,745 cm^−1^. This shift was most prominent in the samples with 5–7 days of exposure. Additionally, all spectra displayed variable but significant amide I and amide II peaks, likely attributed to the protein from cell cortex between the LD and the metasurface. The irregular shape of the amide I peak at 1,655 cm^−1^ is partially explained by its overlap with the H–O–H bending mode of water. LDs displace water in their vicinity, resulting in reduced absorbance from the H–O–H bending mode relative to the bare metasurface used for background measurements.

In the cytoplasmic spectra (Figure 5b), the amide I and II peaks dominate, as expected. However, in samples with the highest ^13^C incorporation (5–7 days), a small peak emerges at 1,615 cm^−1^, attributed to the ^13^C amide I peak [8], [60]. Additionally, the amide II peak exhibits a slight shift of approximately 5 cm^−1^ in these samples, also reflecting the incorporation of ^13^C into amino acids. These observations suggest substantial *de novo* amino acid biosynthesis using glucose as the carbon source.

Glucose metabolism is known to be elevated in adipocyte cells, consistent with their role in lipid storage [61]. Our findings indicate that carbon from glucose metabolism is utilized not only for DNL but also for the synthesis of amino acids. This likely involves the tricarboxylic acid (TCA) cycle, which provides precursors such as α-ketoglutarate for glutamate and oxaloacetate for aspartate [62]. This coupling between the *de novo* synthesis of fatty acids and amino acids highlights a metabolic interdependence that warrants further investigation.

### 3.3 Temporal dynamics of *de novo* lipogenesis

**Figure 6:**
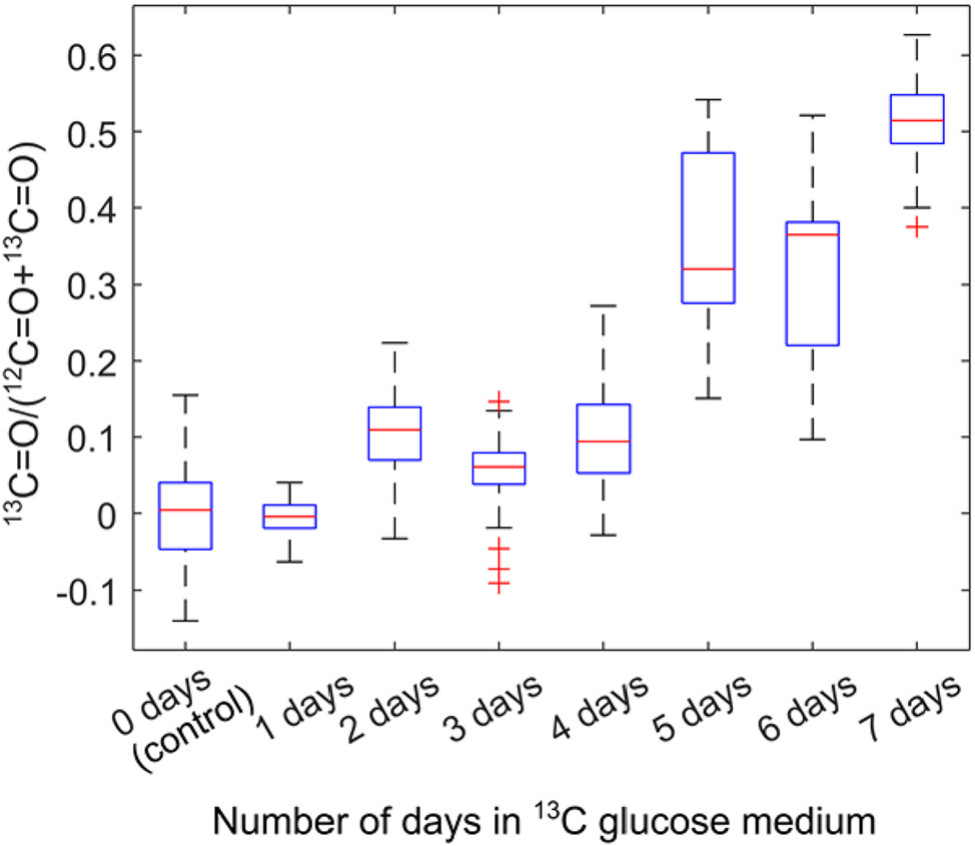
Ratio of ^13^C-labeled lipids to total lipids (^12^C + ^13^C) for different samples with varying exposure time to ^13^C glucose. The horizontal axis represents the number of days in ^13^C glucose, instead of ^12^C glucose, environment, with the total LD maturation time the same across all samples (see Figure 3). The control sample was cultured entirely in ^12^C glucose environment. The intensities of ^13^C and ^12^C lipids were extracted from MIRIAM images, using approximately 100 pixels per sample, selected from regions with the highest ^12^C=O ester intensity. These pixels represent roughly 5–15 LDs from 5 to 9 cells per sample.

To evaluate the temporal dynamics of DNL, we quantified the rate of lipid synthesis by calculating the ratio of the ^13^C=O peak intensity to the total lipid content (defined as the sum of the ^12^C=O and ^13^C=O peak intensities). We note that in most regions of the cells, this ratio is very noisy, since the amount of ^13^C=O and ^12^C=O is too small to be detected outside the LDs (Figure S1). However, this ratio has sufficient signal-to-noise ratio within the LDs. In order to choose the appropriate pixels for analysis, a threshold mask was applied to the ^12^C=O image to identify LDs with a strong ^12^C signal, and the ratio was calculated using only the selected pixels. Approximately 100 pixels were analyzed per sample, representing 5–10 LDs per sample. The results are presented in Figure 6.

The ratio of ^13^C=O to total lipids gradually increased over time, reaching a value of 0.51 ± 0.05 after 7 days of exposure to ^13^C glucose. This ratio is higher than reported in a previous study by Shuster et al. on DNL in 3T3-L1 cells [52], which may be attributed to a number of differences in the experimental protocols. Most importantly, we used longer exposure times (up to 7 days) compared to the 3-day exposure in the earlier study by Shuster et al., which likely resulted in greater ^13^C incorporation in the LDs. Even though the cells have been exposed to ^13^C glucose for a long time (7 days), there is still significant amount of ^12^C=O in the LD. This can be explained by alternate carbon sources that the cells may be using for DNL, which include glutamine and free fatty acids [50], [61], which are not labelled with ^13^C.

Interestingly, the rate of increase in the ^13^C=O ratio appeared to slow down after the first three days. The sample with 4 days of ^13^C glucose exposure exhibited significantly lower ^13^C=O ratios compared to the sample with 7 days of exposure, the difference between the two being that the 4 days sample was in ^12^C-glucose medium for the first three days after the cells were seeded on the metasurface, whereas the 7 days sample was in ^13^C-glucose during this period (see Figure 3). This observation may be explained by a decline in the rate of DNL lipogenesis as LDs mature over time. Alternatively, the observed slowing could be due to an accumulation of ^12^C=O lipids. As the LD size increases, the rate of change in the ^13^C=O-to-total-lipid ratio is expected to decrease even if the absolute rate of ^13^C=O generation remains constant. However, our technique’s limited penetration depth, determined by the plasmonic hotspot, prevents accurate measurement of individual LD sizes, and this hypothesis warrants further investigation.

We also observed considerable variability in the ^13^C=O-to-total-lipid ratio across the samples (as seen from the spread in the data in Figure 6). While Shuster et al. attributed this variability to spatial heterogeneity in ^13^C incorporation [52], we suggest that in our case, this spread is more likely due to experimental error in quantifying the ^13^C ratio. Similar variability was observed across samples with different ^13^C glucose exposure times, which suggests that the variation is not driven by biological differences. Potential sources of error include laser intensity fluctuations, environmental water vapor variability, or inaccuracies in quantifying the ^12^C=O and ^13^C=O intensities caused by spectral artifacts and distortions. Further refinements in experimental design and signal processing may help address these limitations.

## 4 Conclusions

In this work, we utilized MIRIAM, a mid-infrared microscopy technique based on plasmonic metasurfaces, to image and monitor DNL in ^13^C-labeled 3T3-L1 adipocyte cells. ^13^C=O, introduced through ^13^C-labeled glucose, served as a metabolic label for DNL. We demonstrated the incorporation of ^13^C into both proteins and lipids by observing shifts in the amide I/II and C=O vibrational peaks due to ^13^C substitution. As expected, prolonged exposure to ^13^C glucose resulted in greater incorporation of ^13^C=O into LDs.

While glucose metabolism is elevated in adipocyte cells, it is not the sole carbon source for anabolic processes. Other metabolites, such as glutamine and free fatty acids, are also critical contributors to biosynthetic pathways. In this study, only glucose was labeled with ^13^C, providing insights into its role in lipogenesis and protein synthesis. Future studies could expand on this approach by labeling multiple metabolic precursors with IR-active probes, such as ^2^H or azide-containing compounds. This would enable a more comprehensive elucidation of metabolic pathways and provide insights into the interplay between glucose metabolism and alternative anabolic substrates. Such advancements could significantly enhance our understanding of cellular metabolism and its regulation.

## Supplementary Material

Supplementary Material Details
