# Supplementary material for: Combining quantum cascade lasers and plasmonic metasurfaces to monitor de novo lipogenesis with vibrational contrast microscopy
Source: Nanophotonics. 2025 Aug 27;14(23):4133–43. doi: 10.1515/nanoph-2025-0014 (PMC12617835; doi:10.1515/nanoph-2025-0014)
Supplement: Supplementary file 1 — Supplementary Material Details [file j_nanoph-2025-0014_suppl_001.docx]

Supplementary Materials for

Combining quantum cascade lasers and plasmonic metasurfaces to monitor *de novo* lipogenesis with vibrational contrast microscopy

Steven H. Huang *et al.*

This PDF file includes:

Figs. S1 to S2


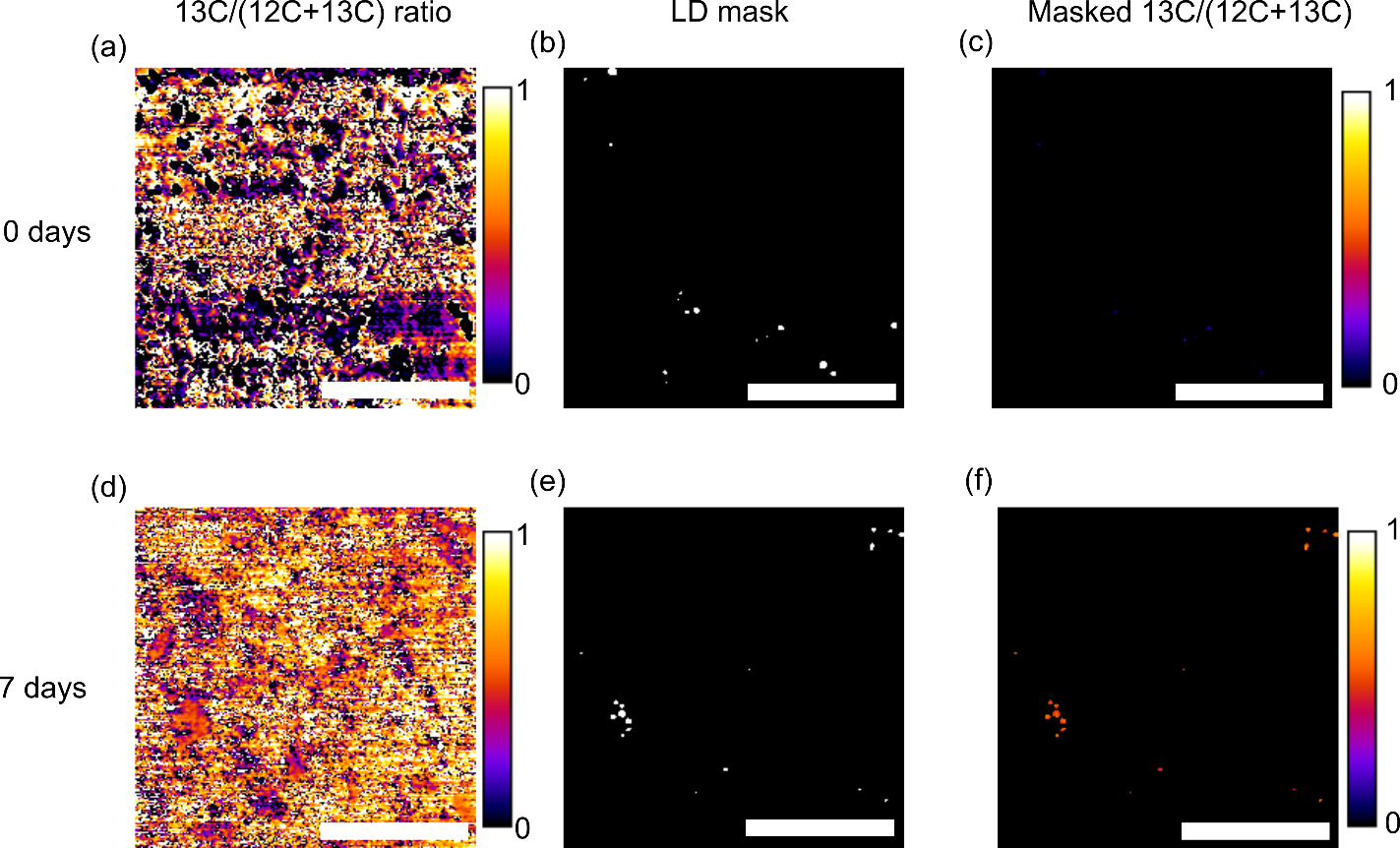


**Figure S1.** Images of ^13^C=O/(^12^C=O+^13^C=O) ratio, for sample with 0-day exposure to ^13^C glucose (a-c) and 7 days exposure to ^13^C glucose (d-f), the same sample as shown in Fig. 4 of the main text. (a), (d) ^13^C=O/(^12^C=O+^13^C=O) ratio image, showing the entire ROI. Outside the LDs, the ratio is extremely noisy because of the small ^12^C=O and ^13^C=O signal. (b), (e) To ensure only LDs with sufficient signal-to-noise ratio are included for further analysis, a mask based on thresholding on ^12^C=O signal is used. (c), (f) Masked ^13^C=O/(^12^C=O+^13^C=O) ratio image. A clear difference in the ^13^C=O level can be seen. Scale bar: 200 μm.


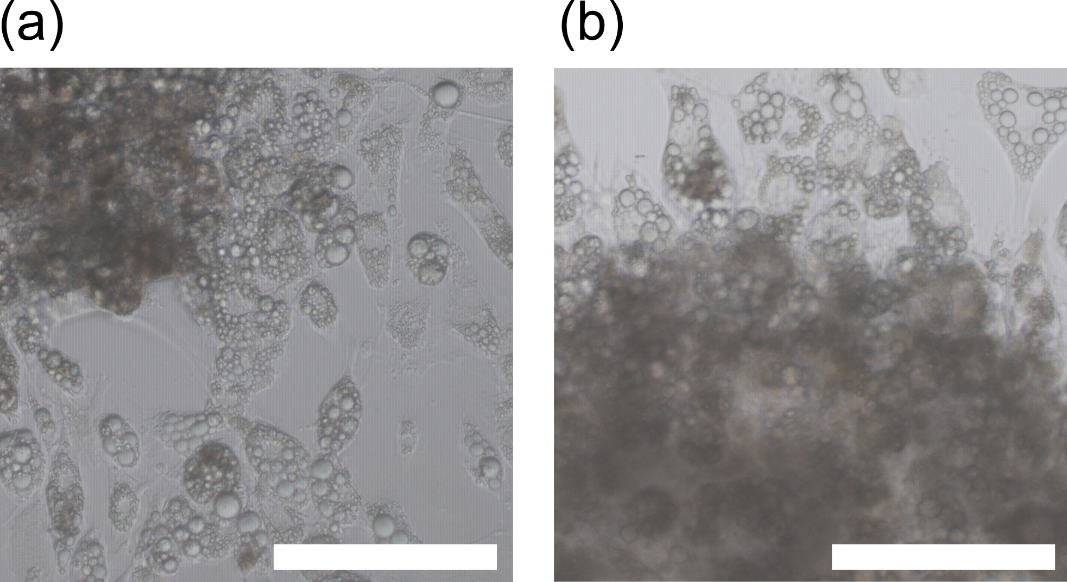


**Figure S2.** Bright-field microscopy images of the samples exposed to ^13^C glucose for 0 days (a) and 7 days (b), corresponding to the images shown in Fig. 4 of the main text. Note that there were some cell aggregates formed on top of the monolayer cell culture for both samples. Scale bar: 200 μm.
